# Cardiovascular Disease Mortality Events and Predictors in the Tehran Lipid and Glucose Study: A 20-Year Cohort Review 

**DOI:** 10.5812/ijem-167662

**Published:** 2026-02-14

**Authors:** Azra Ramezankhani, Fereidoun Azizi, Maryam Barzin, Farzad Hadaegh

**Affiliations:** 1 Prevention of Metabolic Disorders Research Center, Research Institute for Metabolic and Obesity Disorders, Research Institute for Endocrine Sciences, Shahid Beheshti University of Medical Sciences, Tehran, Iran; 2 Endocrine Research Center, Research Institute for Endocrine Disorders, Research Institute for Endocrine Sciences, Shahid Beheshti University of Medical Sciences, Tehran, Iran; 3 Obesity Research Center, Research Institute for Metabolic and Obesity Disorders, Research Institute for Endocrine Sciences, Shahid Beheshti University of Medical Sciences, Tehran, Iran

**Keywords:** Cardiovascular Disease, Tehran Lipid and Glucose Study, Mortality, Risk Factors, Metabolic Syndrome, Predictors

## Abstract

**Context:**

We summarized findings from the Tehran Lipid and Glucose Study (TLGS) on cardiovascular disease (CVD) mortality and its predictors over nearly two decades.

**Evidence Acquisition:**

A review was undertaken to retrieve TLGS publications related to CVD mortality and predictors from the January 1999 to August 5, 2025.

**Results:**

Across TLGS studies, the crude incidence rate of CVD mortality ranged from 1.7 to 4.1 per 1000 person-years in adults aged ≥ 30 years, with higher rates observed among men than women. CVD mortality was associated with traditional risk factors such as diabetes, hypertension, metabolic syndrome (MetS), and dyslipidemia, as well as emerging markers including continuous MetS severity score, anthropometric indices, dynamic weight change, kidney function decline, resting pulse rate, and adherence to cardiovascular health metric. Evidence also highlighted differential associations by age, sex, and diabetes status. Subgroup analyses indicated that elderly participants and those with metabolic abnormalities had higher CVD and sudden cardiac death (SCD) risks.

**Conclusions:**

Tehran Lipid and Glucose Study studies have provided comprehensive insights into the epidemiology and predictors of CVD mortality in the Iranian population, emphasizing both traditional and emerging risk factors. These findings can inform tailored prevention and intervention strategies, though further research incorporating dynamic risk models, omics data, and sex-specific analyses is warranted.

## 1. Context

Cardiovascular disease (CVD) remains the leading cause of death worldwide, accounting for 20.5 million deaths in 2021, with the majority occurring in low- and middle-income countries (1). In the Middle East and North Africa (MENA) region, the burden of CVD has increased due to rapid urbanization, dietary changes, and sedentary lifestyles (2). According to recent estimates, CVD accounts for more than 35 - 45% of total deaths in most MENA countries, with ischemic heart disease and stroke as the primary contributors. Projections suggest that unless major public health interventions are implemented, the CVD mortality burden in the region will continue to rise in the coming decades (3). In Iran, before 2000, comprehensive population-based data on CVD mortality were limited. Early estimates primarily relied on national death registries and hospital-based statistics, which lacked the precision of large-scale epidemiological studies. Nevertheless, these fragmented sources suggested a growing CVD burden, especially in urban areas (4). It was only after 2000 that major initiatives, such as the Iran STEPS (STEPwise Approach to Surveillance) surveys and the establishment of national cohort studies, began to systematically monitor CVD and its risk factors at the population level (5). The most recent comprehensive analysis reports that in 2021, the age-standardized CVD mortality rate in Iran was approximately 255 deaths per 100,000 population (6). This reflects a 43% decrease from 1990, when the rate was about 446 per 100,000. Despite this decline in rates, the absolute number of CVD deaths doubled from about 86,500 in 1990 to nearly 170,000 in 2021 (6). Iran, like many middle-income countries, is undergoing rapid epidemiological, nutritional, and demographic transitions, leading to a sharp increase in modifiable risk factors such as obesity, diabetes, hypertension, and sedentary lifestyles. Ethnic, cultural, and socioeconomic diversity across Iran also contributes to regional disparities in CVD risk and outcomes, underscoring the need for population-specific data (6, 7). Longitudinal cohort studies provide invaluable insights into how these risk factors evolve over time and how they relate to mortality outcomes.

The Tehran Lipid and Glucose Study (TLGS) is a large-scale, ongoing prospective cohort that began in 1999 to investigate the epidemiology and risk factors of non-communicable diseases (NCDs), particularly CVD, among Tehran residents. The study has followed over 15,000 individuals aged ≥ 3 years through multiple phases, collecting detailed lifestyle, metabolic, biochemical, and clinical data via repeated measurements (8). Tehran Lipid and Glucose Study provides a unique opportunity to explore long-term trends and predictors of CVD mortality in a Middle Eastern population. To date, numerous studies using TLGS data have examined CVD mortality rates and associated risk factors. However, a comprehensive review consolidating these findings over the 20-year follow-up period has not yet been conducted. Therefore, this study aims to systematically review and consolidate all published evidence from the TLGS over its 20-year follow-up to: (1) Determine overall and subtype-specific CVD mortality rates in the TLGS cohort; and (2) identify all reported modifiable and non-modifiable risk factors for CVD mortality. In addition, by synthesizing the existing evidence, this review provides a narrative discussion of methodological and knowledge gaps to inform future research directions. This review will significantly enhance our understanding of CVD mortality patterns and predictors in this important Middle Eastern cohort, informing more effective prevention strategies and guiding future research directions.

## 2. Evidence Acquisition

To identify all relevant publications from the TLGS related to CVD mortality and its predictors, a comprehensive literature search was conducted in PubMed, Scopus, and Google Scholar. The search included studies published from January 1999 to August 5, 2025. The search strategy employed a combination of Medical Subject Headings (MeSH) and free-text terms to capture studies on TLGS, cardiovascular mortality, and associated risk factors. Key terms included variations of “Tehran Lipid and Glucose Study” or “TLGS” combined with terms such as “cardiovascular disease”, “CVD”, “myocardial infarction”, “stroke”, “mortality”, “death” and related risk factors like “hypertension”, “diabetes”, and “dyslipidemia”. The literature search was conducted in PubMed, Scopus, and Google Scholar, using tailored strategies: PubMed queries incorporated title/abstract field tags and MeSH terms, while search strings were adapted for Scopus and Google Scholar to match their functionalities. Only English-language original cohort studies were included. Exclusion criteria were: Review articles, editorials, letters, pooled analyses combining TLGS with other cohorts, and studies that did not report effect estimates such as hazard ratios (HR) or odds ratios (OR). Reference lists of eligible studies were screened to identify further pertinent publications. All identified records were imported into a reference management software (e.g., EndNote) to facilitate duplicate removal, title/abstract screening, and full-text review. The specific search terms and strategies used to retrieve articles from each database are presented in Appendix 1 in Supplementary File.

## 3. Results

### 3.1. Overview of Included Studies

A total of 97 records were identified through PubMed, Scopus, and Google Scholar. After removing duplicates and screening titles and abstracts, 57 articles were assessed for eligibility. Following full-text review, a total of 23 eligible publications from the TLGS, spanning 2012 to 2025, were included in this 20-year review. Among the 23 included studies, four examined specific subtypes of CVD mortality including coronary heart disease (CHD) (9), stroke (9), and sudden cardiac death (SCD) (10, 11). Across 23 studies, the sample sizes ranged from approximately 700 to over 10,000 participants, with follow-up durations ranging from approximately 8 to 20 years. Most studies focused on adults aged 30 years and older, while some specifically analyzed subgroups such as elderly populations (12, 13), individuals with diabetes (10, 14), or those with hypertension (15).

### 3.2. Incidence Rate of Cardiovascular Disease Mortality

The included TLGS studies provide long-term population-based estimates of CVD mortality in Iranian adults. A study in 2013, including participants aged ≥ 30 years, reported a crude CVD mortality rate of 4.1 per 1000 person - years (95% confidence interval [CI]: 3.4 - 5.0) in men, compared with 1.7 per 1000 person - years (1.3 - 2.1) in women (16). In another study with adult participants aged ≥ 20 years, participants with type 2 diabetes mellitus (T2DM) experienced a crude CVD mortality rate of 7.9 (6.0 - 10.3) per 1000 person - years (14). A study (11) reported an age - standardized incidence rate of SCD of 2.3 per 1000 person - years (2.1 - 2.7) among participants aged ≥ 30 years. In a recent study (16), 8,241 participants aged ≥ 30 years were followed for a median of 18.1 years, during which 369 CVD deaths occurred, corresponding to a crude incidence rate of 2.88 (2.59 - 3.18) per 1000 person-years.

### 3.3. Major Predictors of Cardiovascular Disease Mortality

A comprehensive review of 23 TLGS publications identified a diverse set of predictors for CVD mortality and its subtypes. These predictors were grouped into six main categories: Diabetes, hypertension, metabolic syndrome (MetS), anthropometric measures, lipid profiles, estimated glomerular filtration rate (eGFR), and miscellaneous factors.

### 3.4. Diabetes

Type 2 diabetes mellitus (T2DM) is a well-established and potent risk factor for CVD and related mortality (17). Evidence from the TLGS demonstrates that both overall CVD mortality and specific outcomes, such as SCD, are substantially higher among individuals with diabetes compared with non-diabetic adults. In a 9-year follow-up study of 9,752 adults ≥ 30 years, individuals with T2DM had more than twice the risk of CVD mortality (hazard ratio [HR]: 2.17; 95% CI: 1.57 - 3.01), compared with non-diabetic participants (18). Similarly, in a long-term study of 8,151 adults aged ≥ 30 years followed for 17.9 years, T2DM was a significant risk factor for SCD, conferring a 2.78-fold higher risk (95% CI: 2.09 - 3.69), compared with non-diabetic individuals (11).

### 3.5. Hypertension

Hypertension is a well-recognized and potent modifiable risk factor for CVD morbidity and mortality (19). Longitudinal evidence from the TLGS demonstrates that elevated blood pressure significantly contributes to the risk of both overall CVD mortality and specific outcomes, including SCD, with particularly pronounced effects in high-risk subgroups such as individuals with T2DM. In a study of 770 diabetic participants (14), hypertension was associated with a 65% higher hazard of CVD mortality (1.65: 0.87 - 3.12), although this did not reach statistical significance. Additionally, hypertension was associated with an increased risk of SCD in 8,151 adults aged ≥ 30 years (1.39: 1.05 - 1.84) (11).

### 3.6. Metabolic Syndrome (MetS)

Metabolic syndrome, encompassing central obesity, hypertension, dysglycemia, and dyslipidemia, is a recognized predictor of CVD (20). Evidence from the TLGS indicates that the association between MetS and CVD outcomes may vary by age, definition criteria, and specific endpoints. In an approximately 9-year follow-up of 7,932 adults aged ≥ 30 years, MetS, as defined by the World Health Organization (WHO), the International Diabetes Federation (IDF), and the Joint Interim Statement (JIS), was not significantly associated with CVD mortality after multivariate adjustment for age, smoking, family history of CVD, intervention, total cholesterol (TC), diabetes medication use, and baseline Body Mass Index (BMI) (21). However, in 922 adults aged ≥ 65 years with a 9.9-year follow-up, MetS was associated with CVD mortality, with the WHO definition showing the strongest association (HR: 1.94; 95% CI: 1.22 - 3.09), followed by the JIS definition (1.65: 1.03 - 2.65), while the IDF definition was not significantly associated (1.37: 0.86 - 2.18) (12). A later study with a 17.9-year follow-up of 5,079 adults ≥ 40 years found that MetS significantly increased the risk of SCD across WHO (HR 1.68, 95% CI 1.20 - 2.35), IDF (1.51: 1.12 - 2.03), and JIS (1.47: 1.08 - 1.98) definitions. Significant individual components included high blood pressure (1.79: 1.29 - 2.48), high fasting plasma glucose (FPG) (1.52: 1.12 - 2.05), and high waist circumference (WC) (1.46: 1.07 - 2.00) (22).

### 3.7. Metabolic Syndrome Severity (cMetS-S)

**Figure 1.**
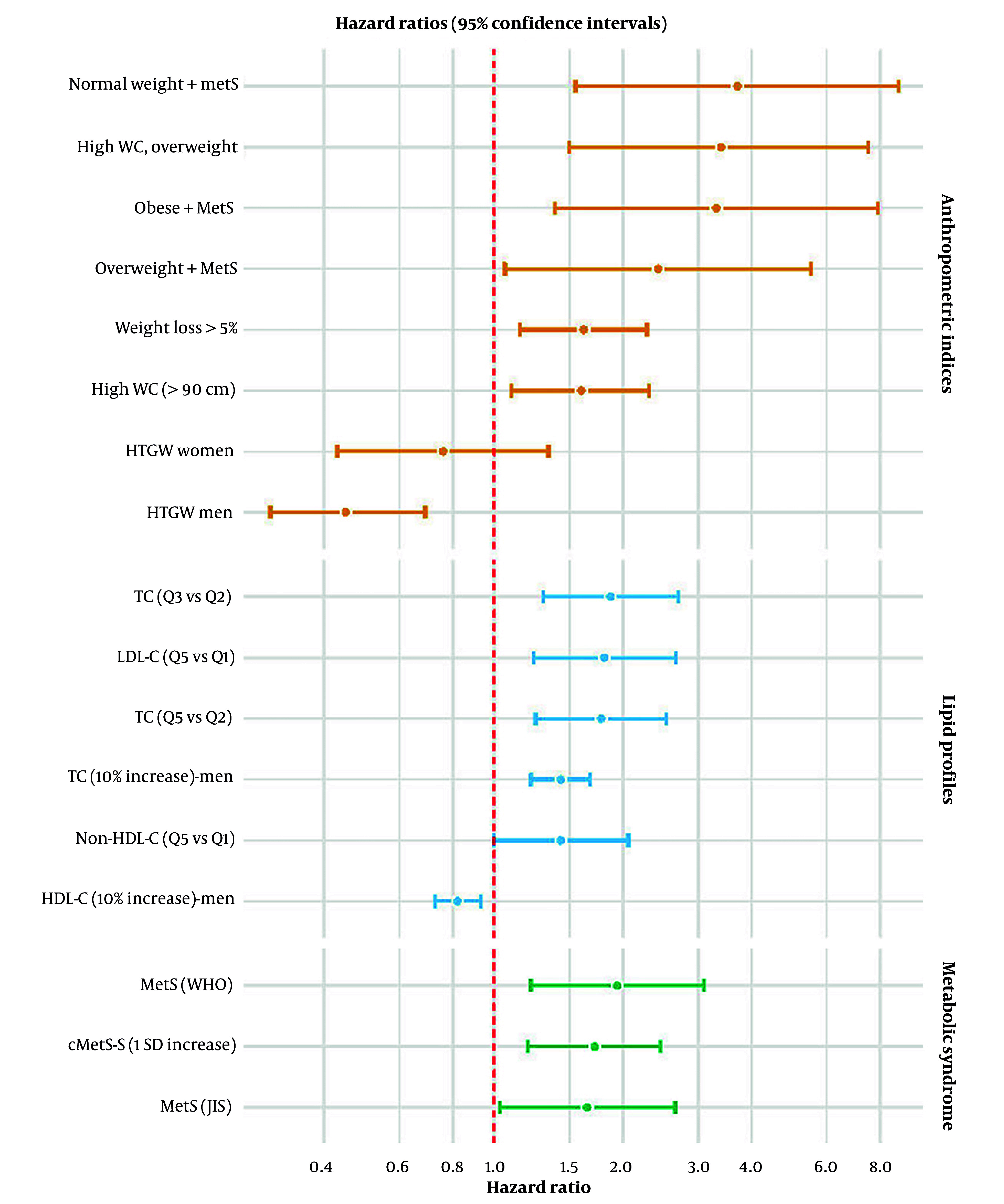
Hazard ratios and 95% confidence intervals for major predictors of cardiovascular disease (CVD) mortality derived from multivariable-adjusted models; Abbreviations: WC, waist circumference; HTGW, hypertriglyceridemic waist; MetS, metabolic syndrome; TC, total cholesterol; LDL-C, low-density lipoprotein cholesterol; HDL-C, high-density lipoprotein cholesterol; Non-HDL-C, non-high-density lipoprotein cholesterol; MetS (WHO), metabolic syndrome defined by World Health Organization criteria; MetS (JIS), metabolic syndrome defined by Joint Interim Statement criteria; cMetS-S, continuous metabolic syndrome score; HR, hazard ratio; CI, confidence interval; SD, standard deviation; Q1, Q2, Q3, Q5, quintiles of exposure distribution.

The continuous metabolic syndrome severity score (cMetS-S), calculated by combining the five key components of MetS, is a standardized, continuous measure of MetS severity. Unlike the traditional binary definition, it quantifies the degree of metabolic risk on a continuum (23). Its association with cardiovascular outcomes has been studied within the TLGS population. In an 18-year follow-up of 8,500 adults aged 20 - 60 years, a 1-SD increase in the age- and sex-specific cMetS-S was associated with a higher risk of CVD mortality (HR: 1.72; 95% CI: 1.20 - 2.45) and SCD (1.60: 1.03 - 2.49) (24). In another study among 7,776 adults aged ≥ 30 years, the optimal cMetS-S cut-off for predicting CVD mortality in the total population was 0.53, with a sensitivity of 51.3% and specificity of 71.9% (25) (Table 1 and Figure 1). 

**Table 1.** Association of Metabolic Syndrome Severity and its Derivatives with Cardiovascular Disease Mortality in Tehran Lipid and Glucose Study

Study, Outcome and Variables	Hazard Ratio (95% CI)
**Mozaffary et al. (** 12 **); (922 adults, ≥ 65 y, 9.9 y follow-up)**	
CVD mortality	
MetS (WHO)	1.94 (1.22 - 3.09)
MetS (JIS)	1.65 (1.03 - 2.65)
MetS (IDF)	1.37 (0.86 - 2.18)
**Masrouri et al. (** 22 **); (5079 adults, ≥ 40 y, 17.9 y follow-up)**	
SCD	
MetS (WHO)	1.68 (1.20 - 2.35)
MetS (IDF)	1.51 (1.12 - 2.03)
MetS (JIS)	1.47 (1.08 - 1.98)
High blood pressure	1.79 (1.29 - 2.48)
High fasting plasma glucose	1.52 (1.12 - 2.05)
High waist circumference	1.46 (1.07 - 2.00)
**Honarvar et al. (** 24 **); (8500 adults, 20 - 60 y, 18 y follow-up)**	
CVD mortality	
cMetS-S; (1 SD increase)	1.72 (1.20 - 2.45)
SCD	1.60 (1.03 - 2.49)
cMetS-S ; (1 SD increase)	

Abbreviations: MetS, metabolic syndrome; cMetS-S, continuous metabolic syndrome severity score; WHO, World Health Organization definition of MetS; IDF, International Diabetes Federation definition of MetS; JIS, joint interim statement definition of MetS; CVD, cardiovascular disease; SCD, sudden cardiac death; SD, standard deviation; 95% CI, 95% confidence interval; TLGS, Tehran lipid and glucose study.

### 3.8. Anthropometric Measures and Derived Indices

Anthropometric measures including BMI, WC, weight, hip circumference (HC), height, and derived indices such as waist-to-hip ratio (WHR) and waist-to-height ratio (WHtR), serve as key indicators of body composition and are widely recognized predictors of cardiovascular risk (26). Evidence from the TLGS reveals variable associations between specific anthropometric parameters and CVD mortality. In a 9.7-year follow-up of 1,199 adults aged ≥ 65 years, obesity phenotypes, defined by different combinations of BMI categories and MetS, were evaluated in relation to incident CVD and mortality. Elderly individuals with MetS had a significantly higher risk of CVD mortality across all BMI categories: Normal weight with MetS (HR: 3.71; 95% CI: 1.55 - 8.85), overweight with MetS (2.42: 1.06 - 5.51), and obese with MetS (3.31: 1.39 - 7.88). In contrast, no significant association was observed among participants without MetS (13). In another study of 881 adults aged ≥ 65 years with a median follow-up of 9.5 years, the impact of general and central adiposity measures (BMI and WC) on CVD mortality was largely explained by traditional risk factors; after adjustment for mediators including hypercholesterolemia, diabetes, and hypertension, no significant associations remained (27). A later study with a 17.9-year follow-up of 10,274 adults (9,089 without T2DM and 1,185 with T2DM), evaluated the associations of anthropometric indices with SCD. Among individuals without T2DM, a 1 - SD increase in WHtR was associated with higher SCD risk (HR: 1.23; 95% CI: 1.00 - 1.50). In participants with T2DM, a 1 - SD increase in WHR conferred higher risk (1.36: 1.05 - 1.76), while a 1 - SD increase in HC was inversely associated with SCD risk (0.75: 0.58 - 0.97) (10). Further evidence came from a study of 8,287 adults aged ≥ 30 years, followed for 17.9 years. High WC (WC > 90 cm in men and women) was associated with increased CVD mortality risk (HR: 1.6; 95% CI: 1.1 - 2.3), with an even stronger effect among overweight individuals (3.4: 1.5 - 7.5) (28). Additionally, in a 17.9 - year follow - up study of 8,151 adults aged ≥ 30 years, high WC (defined as WC ≥ 95 cm for both genders) was a significant predictor of SCD (HR: 1.49; 95% CI: 1.04-2.12) (11). Recent TLGS investigations emphasized dynamic changes in anthropometric measures over time, rather than baseline values alone; in a cohort of 5,436 adults aged ≥ 30 years with a median follow-up of 14.4 years, the impact of three-year weight change on mortality was assessed. A weight loss of more than 5% over three years was associated with an approximately 60% higher risk of CVD mortality (HR: 1.62; 95% CI: 1.15 - 2.28) (29). In a study of 6,834 adults aged ≥ 30 years, followed for a median of 8.8 years, the predictive value of the hypertriglyceridemic waist (HTGW) phenotype, defined as the coexistence of elevated WC and elevated triglycerides (TG), was assessed in relation to CVD incidence and mortality. Compared with non - HTGW individuals, the HTGW phenotype was associated with a lower risk of CVD mortality in men (HR: 0.45; 95% CI: 0.30 - 0.69), whereas no significant association was observed in women (0.76: 0.43 - 1.34) (30) (Table 2 and Figure 1). 

**Table 2.** Association of anthropometric indices and their derivatives with Cardiovascular Disease Mortality in Tehran Lipid and Glucose Study

Study, Outcome and Variables	Hazard Ratio (95% CI)
Mirbolouk et al. (13); (1199 adults, ≥ 65 y, 9.7 y follow-up)	
CVD mortality	
Normal weight + MetS	3.71 (1.55 - 8.85)
Overweight + MetS	2.42 (1.06 - 5.51)
Obese + MetS	3.31 (1.39 - 7.88)
**Moazzeni et al. (** 10 **); (10274 adults, 17.9 y follow-up) – without T2DM**	
SCD	
WHtR; (1 SD increase)	1.23 (1.00 - 1.50)
WHR; (1 SD increase)	1.36 (1.05 - 1.76)
HC; (1 SD increase)	0.75 (0.58 - 0.97)
**Mehran et al. (** 31 **); (8287 adults, ≥ 30 y, 17.9 y follow-up)**	
CVD mortality	
High WC (> 90 cm)	1.6 (1.1 - 2.3)
High WC, overweight	3.4 (1.5 - 7.5)
**Toreyhi et al. (** 11 **); (8151 adults, ≥ 30 y, 17.9 y follow-up)**	
SCD	
High WC (≥ 95 cm)	1.49 (1.04 - 2.12)
CVD mortality	
**Deravi et al. (** 29 **); (5436 adults, ≥ 30 y, median 14.4 y follow-up)**	
Weight loss > 5% over 3 years	1.62 (1.15 - 2.28)
**Samadi et al. (** 30 **); (6834 adults, ≥ 30 y, median 8.8 y follow-up)**	
CVD mortality	
HTGW phenotype men	0.45 (0.30 - 0.69)
Outcome	0.76 (0.43 - 1.34)
HTGW phenotype women	

^z^ Abbreviations: WC, waist circumference; WHR, waist-to-hip ratio; WHtR, waist-to-height ratio; MetS, metabolic syndrome; HTGW, hypertriglyceridemic waist phenotype; CVD, cardiovascular disease; SCD, sudden cardiac death; SD, standard deviation; 95% CI, 95% confidence interval; TLGS, Tehran Lipid and Glucose Study; HC, hip circumference.

### 3.9. Lipid Profiles

Dyslipidemia, marked by elevated TC, low-density lipoprotein cholesterol (LDL-C), and TG alongside reduced high-density lipoprotein cholesterol (HDL-C), contributes critically to CVD pathogenesis by promoting atherosclerosis through endothelial dysfunction, plaque development, and vascular inflammation, thereby increasing the risk of CHD and stroke (32). Evidence from the TLGS underscores the prognostic importance of lipid profiles in CVD mortality. In a study of 4,522 adults aged ≥ 40 years, followed for 15 years, the association of longitudinally measured lipid markers with CHD and stroke mortality was evaluated. During follow-up, 233 CVD deaths (CHD and stroke combined) occurred. In women, each 1 SD increase in TC was associated with a higher risk of CHD mortality (HR: 1.42; 95% CI: 1.07 - 1.89), as was a 1 SD increase in LDL - C (1.41: 1.04 - 1.93) and TG (1.94: 1.02 - 3.75) (9). In another study of 4,148 adults aged ≥ 40 years, followed for 12.4 years, the association of longitudinally measured lipid markers with CVD mortality was evaluated. A total of 233 CVD deaths were observed during the follow-up. The study found that in men, each 10% increase in TC was associated with a 28% higher risk of CVD mortality (HR: 1.28; 95% CI: 1.14 - 1.44). In women, a 10% increase in TC and LDL - C was associated with a significantly higher CVD mortality risk, with HRs of 1.43 (95% CI: 1.22 - 1.68) and 1.21 (1.07 - 1.37), respectively. Conversely, a 10% increase in HDL - C was protective (0.82: 0.73 - 0.93). In the total population, 10% increases in TC and LDL - C corresponded to HRs of 1.30 (1.18 - 1.43) and 1.17 (1.09 - 1.25), respectively (33). In a recent study of 8,241 adults aged ≥ 30 years, followed for 18.1 years, the association of lipid profile measures with CVD and all-cause mortality was evaluated. During follow-up, 369 CVD deaths occurred. For TC, participants in quintile 3 versus quintile 2 (reference) had a higher risk of CVD death (HR: 1.87; 95% CI: 1.30 - 2.70), as did those in quintile 5 versus quintile 2 (1.78; 1.25 - 2.53). For LDL - C, quintile 3 versus quintile 1 (reference) was associated with HR of 1.62 (1.08 - 2.42), and quintile 5 versus quintile 1 with HR of 1.81 (1.24 - 2.66). For non - HDL - C, participants in quintile 5 versus quintile 1 had a higher risk (1.43; 1.00 - 2.06). Analysis of lipid levels treated as continuous variables demonstrated a generally linear relationship with CVD mortality risk, with increased risk observed for all lipid types except TG, for which higher TG levels were associated with a reduced risk of CVD mortality (16) (Table 3 and Figure 1). 

**Table 3.** Association of Lipid Profiles and Their Derivatives with Cardiovascular Disease Mortality in Tehran Lipid and Glucose Study

Study, Outcome and Variables	Hazard Ratio (95% CI)
**Pahlavanzade et al. (** 9 **); (4522 adults, ≥ 40 y, 15 y follow-up) – women**	
CHD mortality	
TC (1 SD increase)	1.42 (1.07 - 1.89)
LDL-C (1 SD increase)	1.41 (1.04 - 1.93)
TG (1 SD increase)	1.94 (1.02 - 3.75)
**Pahlavanzade et al. (** 33 **); (4148 adults, ≥ 40 y, 12.4 y follow-up) – men**	
CVD mortality	
TC (10% increase)	1.28 (1.14 - 1.44)
TC (10% increase)	1.43 (1.22 - 1.68)
LDL-C (10% increase)	1.21 (1.07 - 1.37)
TC (10% increase)	1.30 (1.18 - 1.43)
LDL-C (10% increase)	1.17 (1.09 - 1.25)
HDL-C (10% increase)	0.82 (0.73 - 0.93)
**Ramezankhani et al. (** 16 **); (8241 adults, ≥ 30 y, 18.1 y follow-up)**	
CVD mortality	
TC (Q3 vs Q2)	1.87 (1.30 - 2.70)
TC (Q5 vs Q2)	1.78 (1.25 - 2.53)
LDL-C (Q3 vs Q1)	1.62 (1.08 - 2.42)
LDL-C (Q5 vs Q1)	1.81 (1.24 - 2.66)
**Outcome**	
Non-HDL-C (Q5 vs Q1)	1.43 (1.00 - 2.06)

^z^ Abbreviations: CVD, cardiovascular disease; CHD, coronary heart disease; TC, total cholesterol; LDL-C, low-density lipoprotein cholesterol; HDL-C, high-density lipoprotein cholesterol; TG, triglycerides; Non-HDL-C, non–high-density lipoprotein cholesterol; Q, quintile; SD, standard deviation; 95% CI, 95% confidence interval; TLGS, Tehran lipid and glucose study.

### 3.10. Estimated Glomerular Filtration Rate (eGFR)

Renal dysfunction, assessed by eGFR, is increasingly recognized as an independent risk factor for CVD. Impaired kidney function contributes to CVD pathogenesis through multiple pathways, including fluid overload, hypertension, chronic inflammation, and altered mineral metabolism (34). Evidence from the TLGS underscores the prognostic value of eGFR and its dynamic changes in predicting cardiovascular outcomes. In a study of 2,210 adults aged ≥ 50 years, followed for a median of 14.3 years, the association of kidney function decline with all-cause and CVD mortality was evaluated. During follow-up, 112 CVD deaths occurred. Rapid kidney function decline, defined as an annual eGFR decrease ≥ 3 mL/min/1.73 m² per year, was associated with higher CVD mortality (HR: 1.65; 95% CI: 1.01 - 2.72). Similarly, a ≥ 30% decline in eGFR over six years was associated with increased risk (1.88; 1.09 - 3.23).

### 3.11. Other Predictors

Beyond traditional risk factors, several additional predictors have been identified in the TLGS cohort that provide further insight into mortality outcomes. Factors such as resting pulse rate, the presence of prevalent CVD, and composite measures of ideal cardiovascular health (CVH) have demonstrated significant associations with SCD and mortality. These predictors highlight the importance of both physiological indicators and lifestyle-related health metrics in refining risk stratification for cardiovascular and all-cause mortality. In a 17.9-year follow-up of 8,151 adults aged ≥ 30 years, a resting pulse rate of ≥ 90 beats per minute was associated with a 72% higher risk of SCD (HR: 1.72; 95% CI: 1.22 - 2.42), independent of other factors such as age, sex, CVD history, smoking, WC, hypertension, and diabetes. Additionally, the presence of prevalent CVD increased the risk of SCD by 75% (1.75: 1.26 - 2.45) (11). In another study of 6,388 adults aged ≥ 30 years, followed for a median of 11.3 years, the association between CVH metrics and cardiovascular as well as all - cause mortality was examined. Cardiovascular health was defined according to the American Heart Association’s “Life’s Simple 7,” which includes healthy behaviors (non-smoking, physical activity, healthy diet, normal BMI) and clinical factors (optimal blood pressure, cholesterol, and fasting glucose). During follow-up, 111 SCD occurred, and each additional metric of ideal CVH was associated with a 25% lower risk of SCD (HR: 0.75; 95% CI: 0.64 - 0.88) (35).

## 4. Conclusions

This review summarizes findings from TLGS studies with long-term follow-up, providing robust temporal data on CVD outcomes. It highlights the roles of metabolic factors (diabetes, hypertension, metabolic syndrome [MetS]/continuous metabolic syndrome severity score [cMetS-S]), anthropometric indices, dyslipidemia, kidney function, and lifestyle-related metrics such as CVH. These results reinforce the importance of integrating traditional and emerging risk factors, as well as dynamic changes in health measures, for comprehensive CVD mortality risk assessment and prevention. The findings from the TLGS are consistent with and complemented by other major longitudinal cohorts in Iran. For instance, the Isfahan Cohort Study (ICS) and the Golestan Cohort Study (GCS) have similarly documented the central role of traditional risk factors like hypertension (36) and dyslipidemia (37) in CVD outcomes within their respective populations. Furthermore, the Zanjan Healthy Heart Study reported that MetS was significantly associated with an increased risk of CVD mortality, morbidity, and prolonged hospital stay (38).

However, the TLGS has provided extensive evidence on CVD and its risk factors; with its long-term follow-up spanning up to two decades, it offers robust temporal data that capture the progression of CVD outcomes and their predictors over time. Moreover, the population-based nature of TLGS, including a large and representative sample of Iranian adults, enhances the generalizability of the findings. The precise adjudication of outcomes, particularly CVD mortality, by a dedicated committee is another major strength. This rigorous approach allows validation of death certificates and ensures reliable estimation of cause-specific mortality, which is critical for assessing risk factors accurately (39). Additionally, the studies on TLGS data encompass a comprehensive range of risk factors, including metabolic, anthropometric, biochemical, renal, cardiovascular, and lifestyle-related measures, allowing for a multidimensional understanding of CVD mortality risk. Furthermore, the inclusion of novel and emerging metrics, such as the cMetS-S, dynamic weight change, and composite CVH scores, provides additional depth beyond traditional risk factors. Finally, several TLGS analyses include subgroup-specific assessments by age, sex, and diabetes status, enabling a nuanced understanding of high-risk populations.

However, several gaps remain. Primarily, most studies rely on static, single-time-point measurements, highlighting the need for dynamic models that assess how trajectories of change in key factors over time improve risk prediction. Furthermore, the integration of genetic, proteomic, and metabolomic data is limited, restricting the discovery of novel biomarkers and deeper mechanistic insights. Many analyses combine men and women, emphasizing the need to explore sex-specific pathophysiology to explain differential associations. Statistically, certain investigations, such as those of SCD or specific subgroups, were limited by a low number of events, reducing the precision of risk estimates. Finally, life-course risk modeling remains limited, leaving unanswered questions about how early- or mid-adulthood exposures influence cardiovascular outcomes later in life. Addressing these gaps will further strengthen TLGS’s potential to inform tailored prevention and intervention strategies.

Integrating TLGS data with other major Iranian cohorts, such as ICS (40), GCS (41), and Mashhad Stroke and Heart Atherosclerotic Disorder Study (MASHAD) (42), enables validation of existing CVD mortality prediction models and supports development of a national CVD mortality risk model with broader applicability (43, 44). Such pooled analyses also increase statistical power and allow more precise evaluation of high-risk subgroups. These efforts are being coordinated through the Iran Cohort Consortium, initiated in 2016 to integrate data from multiple large-scale cohorts (45). Furthermore, linkage of the TLGS with the Tehran Cardiometabolic and Genetic Study (TCGS) (46) could substantially expand research potential by merging longitudinal epidemiologic information with detailed genetic and multi-omics datasets. This integration would enable the discovery of novel biomarkers, improve understanding of gene-environment interactions, and enhance precision in cardiovascular risk stratification, thereby supporting the development of tailored prevention and intervention strategies in Iran.

## supplementary material

ijem-24-2-167662-s001.pdf
