# Supplementary material for: Cardiovascular Disease Mortality Events and Predictors in the Tehran Lipid and Glucose Study: A 20-Year Cohort Review
Source: Int J Endocrinol Metab. 2026 Feb 14;24(2):e167662. doi: 10.5812/ijem-167662 (PMC13187687; doi:10.5812/ijem-167662)
Supplement: ijem-24-2-167662-s001.pdf [file ijem-24-2-167662-s001.pdf]

Supplementary File 1

Literature Search Strategy and Included Studies

Databases searched: PubMed, Scopus, and Google Scholar
Timeframe: January 1, 1999- August 5, 2025

1. PubMed Search Strategy

("Tehran Lipid and Glucose Study"[Title/Abstract] OR "TLGS"[Title/Abstract])
AND
("Cardiovascular Diseases"[MeSH] OR "cardiovascular disease"[Title/Abstract] OR "CVD"[Title/Abstract] OR "cardiovascular"[Title/Abstract] OR "heart disease"[Title/Abstract] OR "ischemic heart disease"[Title/Abstract] OR "myocardial infarction"[Title/Abstract] OR "coronary artery disease"[Title/Abstract] OR "heart failure"[Title/Abstract] OR "Stroke"[MeSH] OR "stroke"[Title/Abstract] OR "cerebrovascular"[Title/Abstract])
AND
("Mortality"[MeSH] OR "mortality"[Title/Abstract] OR "death"[MeSH] OR "death"[Title/Abstract] OR "fatal"[Title/Abstract])
AND
("1999/01/01"[Date - Publication] : "2025/08/05"[Date - Publication])

Results: 80 records

2. Scopus Search Strategy

(TITLE-ABS-KEY("Tehran Lipid and Glucose Study") OR TITLE-ABS-KEY(TLGS))
AND
(TITLE-ABS-KEY("cardiovascular disease") OR TITLE-ABS-KEY(CVD) OR TITLE-ABS-KEY(cardiovascular) OR TITLE-ABS-KEY("heart disease") OR TITLE-ABS-KEY("ischemic heart disease") OR TITLE-ABS-KEY("myocardial infarction") OR TITLE-ABS-KEY("coronary artery disease") OR TITLE-ABS-KEY("heart failure") OR TITLE-ABS-KEY(stroke) OR TITLE-ABS-KEY(cerebrovascular))
AND
(TITLE-ABS-KEY(mortality) OR TITLE-ABS-KEY(death) OR TITLE-ABS-KEY(fatal))
AND
(PUBYEAR > 1998 AND PUBYEAR < 2026)

Results: 94 records

3. Google Scholar Search Strategy

(TITLE-ABS-KEY("Tehran Lipid and Glucose Study") OR TITLE-ABS-KEY(TLGS))
AND
(TITLE-ABS-KEY("cardiovascular disease") OR TITLE-ABS-KEY(CVD) OR TITLE-ABS-KEY(cardiovascular) OR TITLE-ABS-KEY("heart disease") OR TITLE-ABS-KEY("ischemic heart disease") OR TITLE-ABS-KEY("myocardial infarction") OR TITLE-ABS-KEY("coronary artery disease") OR TITLE-ABS-KEY("heart failure") OR TITLE-ABS-KEY(stroke) OR TITLE-ABS-KEY(cerebrovascular))
AND
(TITLE-ABS-KEY(mortality) OR TITLE-ABS-KEY(death) OR TITLE-ABS-KEY(fatal))
AND
(PUBYEAR > 1998 AND PUBYEAR < 2026)

Results: 1 additional reference

4. Summary of Records

Database	Records retrieved
PubMed	80
Scopus	94
Google Scholar	1
**Total**	97
